# Association between Dietary Patterns and the Risk of Hypertension among Chinese: A Cross-Sectional Study

**DOI:** 10.3390/nu8040239

**Published:** 2016-04-23

**Authors:** Pei-Fen Zheng, Long Shu, Xiao-Yan Zhang, Cai-Juan Si, Xiao-Long Yu, Wei Gao, Xiao-Qing Tong, Lun Zhang

**Affiliations:** 1 Department of Nutrition, Zhejiang Hospital, Xihu district, Hangzhou 310013, Zhejiang, China; shulong19880920@126.com (L.S.); zxy19740804@sina.com (X.-Y.Z.); xiaosi_32075001@126.com (C.-J.S.); xly2008hi@163.com (X.-L.Y.); gaowei05715133@163.com (W.G.); 3868988899@139.com (X.-Q.T.); zhanglun306@163.com (L.Z.); 2 Department of Digestion, Zhejiang Hospital, Xihu district, Hangzhou 310013, Zhejiang, China

**Keywords:** dietary patterns, hypertension, middle-aged population, factor analysis

## Abstract

Epidemiological studies of different dietary patterns and the risk of hypertension among a middle-aged Chinese population remain extremely scare. Thus, the aim of this study was to identify dietary patterns and investigate the relationship between dietary patterns and the risk of hypertension among Chinese adults aged 45–60 years. The present cross-sectional study includes 2560 participants who reported their dietary intake using a validated food frequency questionnaire (FFQ). Dietary patterns were identified using factor analysis. Anthropometric measurements were obtained using standardized procedures. We used log-binomial regression analysis to examine the associations between dietary patterns and hypertension risk. Four major dietary patterns were identified and labeled as traditional Chinese, animal food, western fast-food, and high-salt patterns. After adjusting for potential confounders, participants in the highest quartile of animal food pattern scores had a greater prevalence ratio (PR) for hypertension (PR = 1.26; 95% confidence interval (CI): 1.064–1.727; *p* < 0.05) in comparison to those from the lowest quartile. Compared with the lowest quartile of high-salt pattern, the highest quartile had a higher prevalence ratio for hypertension (PR = 1.12; 95% CI: 1.013–1.635; *p* < 0.05). Conclusions: Our findings indicated that animal food and high-salt patterns were associated with increased risk of hypertension, while traditional Chinese and western fast-food patterns were not associated with the risk of hypertension. Further prospective studies are warranted to confirm these findings.

## 1. Introduction

Hypertension is one of the leading global public health problems, affecting more than one billion people worldwide [1]. Accompanying the rapid economic development and urbanization that China has experienced, the incidence of hypertension has increased by almost one third from 1991 to 2002, becoming one of the most common chronic diseases [2,3]. In the United States, hypertension is also a significant health problem affecting approximately one-third of the general population [4]. It is well-known that hypertension is a multifactorial chronic disease that may be associated with some factors, such as alcohol consumption, high salt, genetic factors, and dietary factors [5,6].

Over the past few decades, numerous studies have been conducted underscoring the important role of dietary modification in the development of hypertension [7,8,9,10,11]. Nevertheless, the majority of research has been focused on the effect of the intake of individual foods and nutrients on hypertension [7,9,10,11]. Moreover, due to the complexity of the human diet and the potentially synergistic effect of individual foods or nutrients on health, these analyses revealed a limited impact of diet on hypertension [12]. Consequently, dietary pattern analysis has emerged as a multidimensional approach to examine the relationship between diet and the risk of chronic diseases, and it considers the combined effects of foods and potentially facilitates nutritional recommendations [13].

Recently, some studies have shown that there is a growing interest in medical research on the association between dietary patterns and hypertension risk [14,15,16,17]. However, to our knowledge, data on the relationship between dietary patterns and risk of hypertension in the Chinese population are limited [8,17], and are seldom reported among the middle-aged Chinese population. Therefore, the aim of the present study was to derive the major dietary patterns and examine their associations with hypertension risk among a Chinese population aged 45–60 years.

## 2. Subjects and Methods

### 2.1. Study Population

This cross-sectional study was carried out in Hangzhou, the capital of Zhejiang Province, east China, as reported previously [18]. A total of 2734 eligible participants (1440 male, 1294 female) were invited to attend a health examination at the Medical Center for Physical Examination, Zhejiang Hospital, where participants were interviewed face-to-face by a trained interviewer using written questionnaires. 174 participants were excluded because of incomplete anthropometric data, self-reported history of hypertension, or missing or incomplete information on their dietary intake. Finally, 2560 subjects were presented in our analyses. This study was approved by the institutional review and ethics committee of Zhejiang Hospital, and written informed consent was obtained from all participants (2015 tempory audit number (33k)).

### 2.2. Assessment of Dietary Intake

A questionnaire survey was conducted using the 58 food items in a validated semi-quantitative food frequency questionnaire (FFQ) described previously [18]. Briefly, subjects were asked to recall the frequency of consumption of each food item in the previous 12 months and the estimated portion size, using local weight units (e.g., 1 liang = 50 g) or natural units (cups). Consequently, the frequency category (e.g., never, less than 1 time/month, 1 to 3 times/month, 1 to 2 times/week, 3 to 4 times/week, 5 to 6 times/week, 1 time/day, 2 times/day, and 3 times/day) for individual food items was converted into a average daily consumption.

### 2.3. Identification of Dietary Patterns

The Kaiser–Meyer–Olkin Measure of Sample Adequacy and the Bartlett Test of Sphericity were used to assess data adequacy for factor analysis. We used factor analysis (principal component) with varimax rotation to reduce the complexity of dietary patterns. The eigenvalue and scree plot were applied to decide which factors remained [19]. After evaluating the eigenvalues, the scree plot test, and interpretability, factors with eigenvalues ≥2.0 were retained. Individual food items with a factor loading ≥|0.4| were considered to significantly contribute to the pattern in this study. Factor scores were categorized into quartiles (quartile 1 represented a low intake of the food pattern; quartile 4 represented a high intake of the food pattern).The labeling of dietary patterns was based on the interpretation of foods with high factor loadings for each dietary pattern [20].

Dietary patterns were identified by factor analysis, using standard principal component analysis as reported previously for this study population [18]. The traditional Chinese pattern was characterized to have high loadings of foods such as rice, steamed bun/noodles, coarse grains, tubers, fresh vegetables and fruits, fish and shrimp, miscellaneous bean, and tea. The animal food pattern was characterized by high consumption of rice, mushroom, red meat, fish and shrimp, seafood and fats/oils. The western fast-food pattern was characterized by high consumption of fast foods, snacks, chocolates, coffee, and drinks. The high-salt pattern was characterized to have high loadings of pickled vegetables, processed and cooked meat, bacon and salted fish and bean sauce.

### 2.4. Blood Pressure Measurement

Blood pressure was measured after ten minutes in the sitting position with a standard mercury sphygmomanometer by a trained nurse. Then, blood pressure was measured three times, and the mean of three measurements was considered as the subject’s blood pressure in our analyses.

### 2.5. Assessment of other Variables

The physical activity levels were assessed with the International Physical Activity Questionnaire (IPAQ).The results were expressed as metabolic equivalents in hours per week (MET-h/week). Information on smoking status comprised the categories of never smokers, current smokers, and former smokers. Education level was categorized in three classes: primary school or below, middle and high school, and junior college or above, as described elsewhere [18]. Total energy intake was estimated through the FFQ, expressed in kilocalories per day (kcal/day) and categorized according to quartile.

### 2.6. Definition of Hypertension

Hypertension was defined as a systolic pressure of 140 mmHg or higher and/or a diastolic pressure of 90 mmHg or higher [21].

### 2.7. Statistical Analyses

The general characteristics of study participants across quartiles categories of each dietary pattern scores were calculated. Data are presented as mean ± SD for continuous variables or sum (percentages) for categorical variables. We used analysis of variance (ANOVA) to describe mean differences in continuous variables and the chi-squared test to assess the difference in categorical variables. Analysis of covariance was used to compare the difference of systolic blood pressure (SBP) and diastolic blood pressure (DBP) in the highest categories compared with the lowest categories of four dietary patterns. After adjusting for age, smoking status, economic income, educational level, physical activity level, body mass index, and total energy intake, log-binomial regression analysis was used to investigate the relationship between dietary patterns and the risk of hypertension. All statistical analyses were performed using the SPSS software package version 20.0 for Windows (SPSS Inc., Chicago, IL, USA). Two-sided *p*-values < 0.05 were considered statistically significant.

## 3. Results

The overall prevalence of hypertension in our study population was 25.1%. Demographic and lifestyle characteristics of participants organized by gender are presented in Table 1 (*n* = 2560). There were no significant differences between participants by age, education level, economic income, physical activity, and the prevalence of hypertension. In addition, we found significant differences between participants by smoking status, and the prevalence of obesity.

Four major dietary patterns were identified, namely traditional Chinese, animal food, western fast-food, and high-salt patterns, which explained 7.2%, 7.5%, 7.2% and 6.0% of the dietary intake variance, respectively. The factor loading matrix of these patterns have been described in a previous study [18]. The general characteristics of study participants across quartile categories of each dietary pattern scores are presented in Table 2. Subjects in the fourth quartile of the traditional Chinese pattern were more likely to be female, older, and had lower prevalence of obesity and hypertension, and higher income. Compared with those in the lowest quartile, individuals in the highest quartile of the animal food pattern were more likely to be male, smoker, younger, and had higher prevalence of obesity and higher economic income. Moreover, subjects who belonged to the highest quartile of western fast-food pattern were more likely to be female, younger, and had higher income than those in the lowest quartile. Subjects in the highest quartile of high-salt pattern were more likely to be smokers, male with lower education level and economic income than those in the lowest quartile.

Table 3 shows the difference of SBP and DBP by quartile (Q) categories of dietary pattern scores using an analysis of covariance model. After adjusting for gender, age, smoking status, economic income, educational level, physical activity level, body mass index, and total energy intake, participants in the highest quartile of high-salt pattern had higher SBP and DBP (Q1: 127.05 ± 18.21 *vs*. Q4: 132.30 ± 17.49; Q1: 77.32 ± 12.93 *vs*. Q4: 82.57 ± 13.04, *p* < 0.05). Subjects in the highest quartile of animal food pattern had a higher SBP than those in the lowest quartile (Q1: 129.15 ± 18.47 *vs*. Q4: 132.28 ± 18.15, *p* < 0.05). In addition, those participants in the highest quartile of the traditional Chinese pattern had lower SBP than those in the lowest quartile (Q1: 133.51 ± 19.01 *vs*. Q4: 128.69 ± 16.70, *p* < 0.05).

The relationship between dietary patterns and hypertension by log-binomial regression analysis was shown in Table 4. Before adjustment for potential confounders, compared with those in the lowest quartile, individuals in the highest quartile of the traditional Chinese pattern had a lower prevalence ratio for hypertension (PR = 0.93; 95% CI: 0.531–0.982, *p* < 0.05); After adjustment for potential confounders, subjects in the highest quartile of animal food and high-salt pattern scores had a higher likelihood of hypertension (PR = 1.37, 95% CI: 1.064–1.727; PR = 1.12, 95% CI: 1.013–1.635 respectively, *p* < 0.05) those in the lowest quartile. However, the western fast-food pattern showed no association with the risk of hypertension.

## 4. Discussion

In this study population, four dietary patterns were identified: traditional Chinese, animal food, western fast-food, and high-salt patterns. The results of this study indicate that animal food and high-salt patterns are associated with increased risk of hypertension, while the traditional Chinese and western fast-food pattern were not associated with the risk of hypertension in a middle-aged Chinese population. Notably, to our knowledge, this is the first study from a middle-aged Chinese population to report the associations between different dietary patterns and hypertension risk.

In this study, we found a positive relationship between the animal food pattern and hypertension risk. Our findings were consistent with previous studies, which reported a significant association between animal food and the risk of hypertension [22,23]. The detrimental effect of the animal food pattern could be attributable to this pattern’s unhealthy constituents (e.g., red meat and fats/oils). High consumption of meat, especially red meat containing high amounts of saturated fat and cholesterol, was associated with an elevated risk of obesity [24]. A substantial body of evidence has demonstrated that obesity may play a key role in the development of hypertension [25]. Additionally, the positive association also may be related to a lower level of physical activity in this pattern. A recent systematic review and meta-analysis of physical activity and hypertension risk concluded that the level of physical activity was significantly associated with the risk of hypertension [26]. Furthermore, higher meat consumption may reflect some undetected dietary behavior or lifestyle contributing to a rise in blood pressure.

The high-salt pattern was traditionally considered to be an unhealthy pattern, which was characterized by a high consumption of pickled vegetables, processed meat, cooked meat, bacon, salted fish, and bean paste. We found a positive association between this pattern and hypertension risk. There are several possible explanations for the positive association between high-salt pattern and the risk of hypertension. Firstly, salt intake has been acknowledged as a direct risk factor for hypertension [27]. Secondly, in this pattern, meat products, including processed and cooked meat, are a major source of saturated fat and cholesterol, which may increase the risk of obesity, an important risk factor for hypertension [24,25]. Thirdly, results from this study indicate that subjects who belonged to the fourth quartile of high-salt pattern have a lower physical activity level, compared to those in the lowest quartile. Emerging evidence has shown that physical activity as a form of energy expenditure is closely related to blood pressure [28].

The traditional Chinese pattern identified in our analyses was not associated with the risk of hypertension. Our findings were inconsistent with previous studies [5,8], suggesting that the traditional Chinese pattern was associated with a reduced risk of hypertension. There are several possible explanations for this null association. Firstly, a previous large-scale intervention study on Dietary Approaches to Stop Hypertension (DASH) revealed that a dietary pattern rich in fruits and vegetables could decrease the risk of hypertension [29]. Besides, some studies have also indicated that antioxidants (e.g., Vitamins C and E) abundant in fruits and vegetables can help to prevent oxidative stress [30,31]. However, in contrast to Western populations which often consume raw and fresh vegetables, Chinese populations tend to consume more cooked vegetables, which may result in the loss of some antioxidant content [17]. Secondly, the middle-aged Chinese population, especially southern Chinese, often like to consume cooked vegetables with salt or pickled (as discussed in our previous study) [18]. It is well-known that a high intake of salt is positively associated with the risk of hypertension [27]. Finally, a null association between this pattern and hypertension risk could also be due to reverse causality. Participants with risk of hypertension may be advised to change dietary habits during a routine examination; e.g., limiting the intake of salt. In a word, these possibilities could not be excluded in this study.

In our analyses, we did not find a significant association between western fast-food pattern and hypertension risk. Our results were not in agreement with previous studies, which demonstrated the significant relationship between high-fast food consumption and the risk of hypertension [32]. To our knowledge, the inconsistent findings could be attributed to the difference in this pattern's constituents. Compared with the western pattern (having high consumption of refined grains, red meat, butter, high-fat dairy products, sweets and desserts, pizza and soft drinks) [33], the western fast-food pattern in the present study is characterized by high consumption of fast foods, snacks, chocolates, coffee, and drinks, and low consumption of red meat, butter, and high-fat dairy products. Moreover, the study participants were predominately a group of Chinese population aged 45–60 years, who consumed large amounts of tea. There is a wealth of evidence suggesting that drinking green tea is associated with a reduced risk for obesity and related chronic diseases [34,35], which is considered as an important risk factor for hypertension. Furthermore, recent a systematic review and meta-analysis of tea consumption and the risk of hypertension indicated that drinking tea was associated with a decreased risk of hypertension [36].

The prevalence of hypertension in this study is 25.1%. Similarly, Qin *et al*. [17] reported the prevalence of hypertension in Jiangsu Province is 26.7%. Moreover, a previous study conducted in Pakistani urban adults reported that 33% of the adult population suffers from hypertension [37]. Furthermore, in the United States, approximately one-third of the general population is suffering from hypertension, which has become significant health problem [4]. Over the past decades, dietary patterns are changing rapidly worldwide, and the prevalence of some chronic non-communicable diseases (e.g., hypertension, diabetes) which are associated with diet are increasing rapidly. Thus, it makes sense to elucidate the relationship between dietary patterns and the risk of chronic diseases and provide a scientific rationale for formulating dietary guidelines.

## 5. Strengths and Limitations

The present study holds several strengths and limitations. First, to our knowledge, this is the first study reporting the relationship between dietary patterns and hypertension risk among a middle-aged Chinese population. It provides important information on the role of dietary modifications in the prevention of hypertension in a middle-aged Chinese population. Second, we evaluated dietary intakes using a validated semi-quantitative FFQ by a face-to-face interview. This tool enabled us to capture more reliable information on participants’ dietary intakes. Besides, we have adjusted for potential known confounding variables in our analyses. Nonetheless, some limitations should be considered when interpreting our findings. First, this study was cross-sectional in nature, and the causal relationship between dietary patterns and hypertension could not be determined. Thus, our findings need to be confirmed in a future prospective study. Second, several subjective or arbitrary decisions (e.g., decision to include the variables in the patterns) in the use of factor analysis need to be considered [38]. Finally, the study participants were volunteers recruited from a health screening examination in the city of Hangzhou, Zhejiang Province, China. Therefore, our results may not be generalized to the general population.

## 6. Conclusions

In summary, our results indicate that animal food and high-salt patterns are associated with a higher risk of hypertension among a middle-aged Chinese population. Our findings provided additional support for such dietary recommendations for the prevention of hypertension among a middle-aged Chinese population. However, larger prospective studies are required to confirm our findings and identify the clear associations between dietary patterns and hypertension risk.

## Figures and Tables

**Table 1:** Demographic and lifestyle characteristics of participants by gender in Hangzhou.

Variables	Male (*n* = 1336)	Female (*n* = 1224)	*p*-Value
Demographic			
Age (years)	51.80 ± 4.53	50.96 ± 4.80	*p* = 0.653
Smoking status (%)			
Never	890 (66.6)	1054 (86.1)	*p* = 0.000
Former	36 (2.7)	8 (0.7)	
Current	410 (30.7)	162 (13.2)	
Education (%)			
<High school	293 (21.2)	258 (21.1)	*p* = 0.781
High school	434 (31.0)	392 (32.0)	
>High school	609 (47.8)	574 (46.9)	
Monthly income per person (%)			
≤2000 (RMB)	355 (26.6)	361 (29.5)	*p* = 0.093
2000–3000 (RMB)	560 (41.9)	464 (37.9)	
>3000 (RMB)	421 (31.5)	399 (32.6)	
Physical activity (%)			
Light	1100 (82.3)	985 (80.5)	*p* = 0.058
Moderate	206 (15.4)	192 (15.7)	
Vigorous	30 (2.3)	47 (3.8)	
Hypertension	349 (26.1)	293 (23.9)	*p* = 0.203
Obese (%)	166 (12.4)	155 (5.4)	*P* = 0.001

Categorical variables are presented as sum and percentages, and continuous variables are presented as Mean ± SD; * *p*-values for continuous variables (Analysis of variance) and for Categorical variables (chi-square test).

**Table 2:** The general characteristics of the study participants across quartile (Q) categories of dietary pattern scores in the Hangzhou.

	Traditional Chinese		* *p*	Animal Food		* *p*	Western Fast-Food		* *p*	High-Salt		* *p*
	Q1 (*n* = 640)	Q4 (*n* = 640)		Q1 (*n* = 640)	Q4 (*n* = 640)		Q1 (*n* = 640)	Q4 (*n* = 64)		Q1 (*n* = 640)	Q4 (*n* = 640)	
Age (year)	50.0 ± 0.2	51.9 ± 0.3	<0.001	51.8 ± 0.3	50.3 ± 0.2	<0.001	51.5 ± 0.2	49.7 ± 0.2	<0.001	50.7 ± 0.2	51.0 ± 0.2	0.789
Gender (%)			<0.001			<0.001			<0.001			<0.001
Male	543 (84.8)	208 (32.5)		328 (51.2)	208 (32.5)		433 (67.6)	336 (52.5)		323 (50.5)	413 (64.5)	
Female	97 (15.2)	432 (67.5)		312 (48.8)	432 (67.5)		207 (32.4)	304 (47.5)		317 (49.5)	227 (35.5)	
Obesity (%)	113 (17.6)	53 (8.3)	<0.001	65 (10.1)	108 (16.8)	<0.001	99 (15.5)	68 (10.7)	0.01	79 (12.3)	95 (14.9)	0.374
Hypertension (%)	261 (40.8)	176 (27.5)	<0.001	196 (30.7)	223 (34.9)	<0.001	210 (32.8)	162 (25.3)	0.035	171 (26.7)	215 (33.6)	0.030
Smoking status (%)			<0.001			<0.001			0.708			0.011
Current	319 (49.9)	68 (10.7)		121 (18.9)	253 (39.5)		176 (27.5)	184 (28.8)		148 (23.2)	195 (30.4)	
Former	5 (0.8)	5 (0.8)		7 (1.1)	11 (1.7)		4 (0.6)	6 (0.9)		6 (0.9)	7 (1.1)	
Never	316 (49.3)	567 (88.5)		512 (80.0)	376 (58.8)		460 (71.9)	450 (70.3)		486 (75.9)	438 (68.5)	
Educational level (%)			0.551			<0.001			0.588			<0.001
<High school	162 (25.3)	143 (22.4)		230 (36.0)	91 (14.2)		152 (23.7)	133 (20.8)		116 (18.1)	177 (27.7)	
High school	200 (31.2)	196 (30.7)		222 (34.7)	172 (26.9)		191 (29.9)	191 (29.9)		189 (29.6)	203 (31.7)	
>High school	278 (43.5)	301 (46.9)		188 (29.3)	377 (58.9)		297 (46.4)	316 (49.3)		335 (52.3)	260 (40.6)	
Average monthly income per person (%)			<0.001			<0.001			<0.001			<0.001
≤2000 (RMB)	218 (34.1)	160 (25.0)		261 (40.8)	111 (17.3)		207 (32.4)	141 (22.1)		123 (19.2)	225 (35.2)	
2000–3000 (RMB)	257 (40.1)	245 (38.3)		268 (41.9)	244 (38.1)		269 (42.1)	219 (34.2)		244 (38.2)	253 (39.5)	
>3000 (RMB)	165 (25.8)	235 (36.7)		111 (17.3)	285 (44.6)		164 (25.5)	280 (43.7)		273 (42.6)	162 (25.3)	
Physicalactivity (%)			0.116			<0.001			0.550			0.346
Light	506 (79.1)	535 (83.6)		452 (70.7)	568 (88.8)		517 (80.8)	532 (83.1)		543 (84.8)	516 (80.7)	
Moderate	106 (16.6)	95 (14.9)		143 (22.3)	66 (10.3)		103 (16.1)	96 (15.0)		76 (11.9)	102 (15.9)	
Vigorous	28 (4.3)	10 (1.5)		45 (7.0)	6 (0.9)		20 (3.1)	12 (1.9)		21 (3.3)	22 (3.4)	
Total energy intake (Kcal/day)	1830.6 ± 323.5	1631.6 ± 224.21	<0.001	1707.1 ± 254.8	1757.6 ± 289.0	0.457	1738.4 ± 269.7	1724.5 ± 311.8	0.513	1620.7 ± 224.8	1840.2 ± 320.0	<0.001

Categorical variables are presented as sum and percentages, and continuous variables are presented as Mean ± SD (standard deviation); * *p* values for continuous variables (analysis of variance) and for categorical variables (chi-square test); *p* *<* 0.05 was considered statistically significant. Monthly income per person (RMB) was presented as mean.

**Table 3:** Analysis of covariance model to evaluate the difference of SBP and DBP by quartile (Q) categories of dietary pattern scores.

	SBP (mmHg)	*p*	DBP (mmHg)	*p*
Traditional Chinese pattern				
Q1 (*n* = 640)	133.51 ± 19.01	<0.05	83.90 ± 14.58	0.070
Q4 (*n* = 640)	128.69 ± 16.70		79.73 ± 12.80	
Animal food pattern				
Q1 (*n* = 640)	129.15 ± 18.47	<0.05	81.11 ± 12.33	0.133
Q4 (*n* = 640)	132.28 ± 18.15		83.06 ± 14.52	
Western fast-food pattern				
Q1 (*n* = 640)	132.39 ± 17.82	0.147	82.17 ± 13.70	0.259
Q4 (*n* = 640)	129.68 ± 18.84		79.89 ± 13.85	
High-salt pattern				
Q1 (*n* = 640)	127.05 ± 18.21	<0.05	77.32 ± 12.93	<0.05
Q4 (*n* = 640)	132.30 ± 17.49		82.57 ± 13.04	

Adjusted for gender, age, physical activity, smoking status, economic income, educational level, body mass index, and total energy intake. Abbreviation: SBP, Systolic blood pressure; DBP, Diastolic blood pressure. *p* *<* 0.05 was considered statistically significant.

**Table 4:** Multivariable adjusted prevalence rate ratio (95% CI) for hypertension across the quartile (Q) categories of dietary pattern scores.

	Traditional Chinese Pattern Score			Animal Food Pattern Score			Western Fast-Food Pattern Score			High-Salt Pattern Score		
	Q1	Q4	*p*	Q1	Q4	*p*	Q1	Q4	*p*	Q1	Q4	*p*
Hypertension												
Model 1	1.00	0.93 (0.531, 0.982)	0.044	1.00	1.69 (1.243,2.175)	0.004	1.00	0.77 (0.603,1.191)	0.436	1.00	1.89 (1.409, 2.592)	0.000
Model 2	1.00	1.21 (0.846, 1.747)	0.756	1.00	1.35 (1.137, 1.905)	0.032	1.00	1.08 (0.651, 1.724)	0.661	1.00	1.54 (1.201, 2.485)	0.008
Model 3	1.00	1.14 (0.760, 1.652)	0.627	1.00	1.26 (1.064, 1.727)	0.045	1.00	1.36 (0.995, 1.825)	0.777	1.00	1.12 (1.013, 1.635)	0.046

Model 1: unadjusted; Model 2: further adjusted for gender, age, physical activity level; Model 3: additionally adjusted for body mass index, total energy intake; Q4: the highest quartile of dietary patterns; Q1: the lowest quartile of dietary patterns (reference); CI: confidence interval.
